# From Plant to Waste: The Long and Diverse Impact Chain Caused by Tobacco Smoking

**DOI:** 10.3390/ijerph16152690

**Published:** 2019-07-28

**Authors:** Maria Christina B. Araújo, Monica F. Costa

**Affiliations:** 1 Departamento de Oceanografia e Limnologia, Universidade Federal do Rio Grande do Norte, Via Costeira S/N, Praia de Mãe Luíza, Natal 59014-100, Brazil; 2 Departamento de Oceanografia, Universidade Federal de Pernambuco, Av. Arquitetura, s/n, Recife 50740-550, Brazil

**Keywords:** plastic pollution, cigarette butts, environmental impacts, tobacco smoking

## Abstract

Smoking is a social phenomenon of global scope. The impacts start from the cultivation of the plant to the disposal of cigarette butts in the most diverse places. These aspects go beyond economic and public health issues, also affecting natural environments and their biota in a serious and indistinct way. Of the six trillion cigarettes consumed globally each year, four and a half trillion are disposed somewhere in the environment. Cigarette butts are predominantly plastic, non-biodegradable waste, prevalent in coastal environments in various parts of the world, and with high potential for generating impacts on a wide range of socioeconomic and environmental aspects. Among the 5000 compounds found in a cigarette, those with higher toxic potential are mainly concentrated in the filter and in tobacco remnants, which are items found in discarded cigarette butts. After surveying published studies on this topic, the present study addressed the interaction between the impacts related to tobacco smoking, highlighting the problem as an important and emerging issue that demands joint efforts, and actions especially focused on the reduction of environmental impacts, an aspect that has not yet been assessed.

## 1. Introduction

Cigarettes usually have two basic components: processed tobacco and a synthetic filter (both wrapped in paper). More than 5000 compounds can be found in their composition, of which at least 150 have been recognized as highly toxic, due to their carcinogenic and mutagenic potential [1,2,3]. The compounds with higher toxic potential are mainly concentrated in the remnants of tobacco and the filters after consumption, which are exactly the items that compose the discarded cigarette butts [3,4]. Cigarette butts are predominantly plastic, non-biodegradable waste, prevalent in coastal environments in various parts of the world, and with high potential for generating impacts on a wide range of socioeconomic and environmental aspects. Filters have been incorporated into cigarettes since 1950, in an attempt to protect smokers from the damages caused by the inhalation of tobacco smoke. Over the last 50 years, 99% of smokers have started to consume filtered cigarettes [5]. According to Harris [6], the only thing achieved by the filters was the spread of the smoking habit, increasing both the risk of smokers’ dependence and the load of non-biodegradable and toxic synthetic waste in the environment.

The material that compose the filters (cellulose acetate) is a synthetic polymer made from cellulose (natural compound of vegetable origin) through the addition of acetic anhydride and acetic acid (a process known as acetylation), in addition to plasticizing compounds. As a result of this composition, cigarette butts are classified as a plastic item in most of the numerous publications related to marine litter in the world. However, more recently, cigarette filters began to be considered an isolated category, mainly due to the amount found [7]. Unlike cellulose, which can be biodegraded by several organisms that produce the cellulose enzyme, cellulose acetate has a limited potential for biodegradation, because it is a synthetic product obtained from the modification of cellulose by chemical processes. Although it is photodegradable, its disintegration is probably hampered by high fiber compaction and addition of plasticizers [8]. 

Substances that make cigarettes potentially hazardous to smokers (active and passive) and potentially harmful to the environment are incorporated throughout the production process, from tobacco planting to the final product for sale [2,9]. In the cigarette making process, dozens of substances (such as flavorings, solvents, preservatives, plasticizers, and other additives) are incorporated into the tobacco, the filters, and the enclosures [10,11]. The application of pesticides, insecticides, herbicides, and fungicides insert metals into the plant (*Nicotiana tabacum*), which are especially accumulated in the leaves [12,13,14]. According to Tso [15], the existence of metals in cigarettes can also be attributed to the tobacco growing process, given that the plant easily accumulates metals retrieved from the underlying soil.

In the smoking context, i.e., at all stages, involving tobacco planting, cigarette preparation, consumption, and disposal of cigarette butts, there is a strong influence on socioeconomic and environmental aspects, with numerous and wide impacts on human health and the environment.

## 2. Associated Socioeconomic and Environmental Impacts

The popularization of cigarette consumption took place in the second half of the 19th century, to some extent stimulated by urbanization and the fast pace of city life. However, since the 1950s, consumption has intensified due to the incentive provided by large cigarette companies through mass media, with campaigns initially associated with the American cowboy’s way of life and values. Later, consumption was promoted by advertisements of individuals practicing extreme sports and smoking, in an attempt to associate smoking with independence and freedom [16]. Although this type of advertising has been abolished almost completely nowadays, the smoking habit has remained strongly established. In 2016, for example, 5505 trillion cigarettes were consumed worldwide [17]. This is probably due to the fact that cigarettes are considered a licit drug and are therefore easy to obtain, including by means of smuggling.

Spending on treatments for smoking-related diseases has often been higher than the amount collected with taxes charged to cigarette marketing. In Brazil, for example, the consumption of cigarettes has caused a loss of 56.9 billion real (the Brazilian currency) to the country each year. Of this total, 39.4 billion real were spent on direct medical costs, and 17.5 billion real on indirect costs, resulting from loss of productivity caused by premature deaths, or loss of workers’ capacity. On the other hand, the country annually collects only 13 billion real in taxes charged on the sale of cigarettes, i.e., this amount only represents 23% of the expenses related to the damages caused by the tobacco epidemic. The remaining money required is taken from resources that could be allocated to other areas [18].

**Figure 1:**
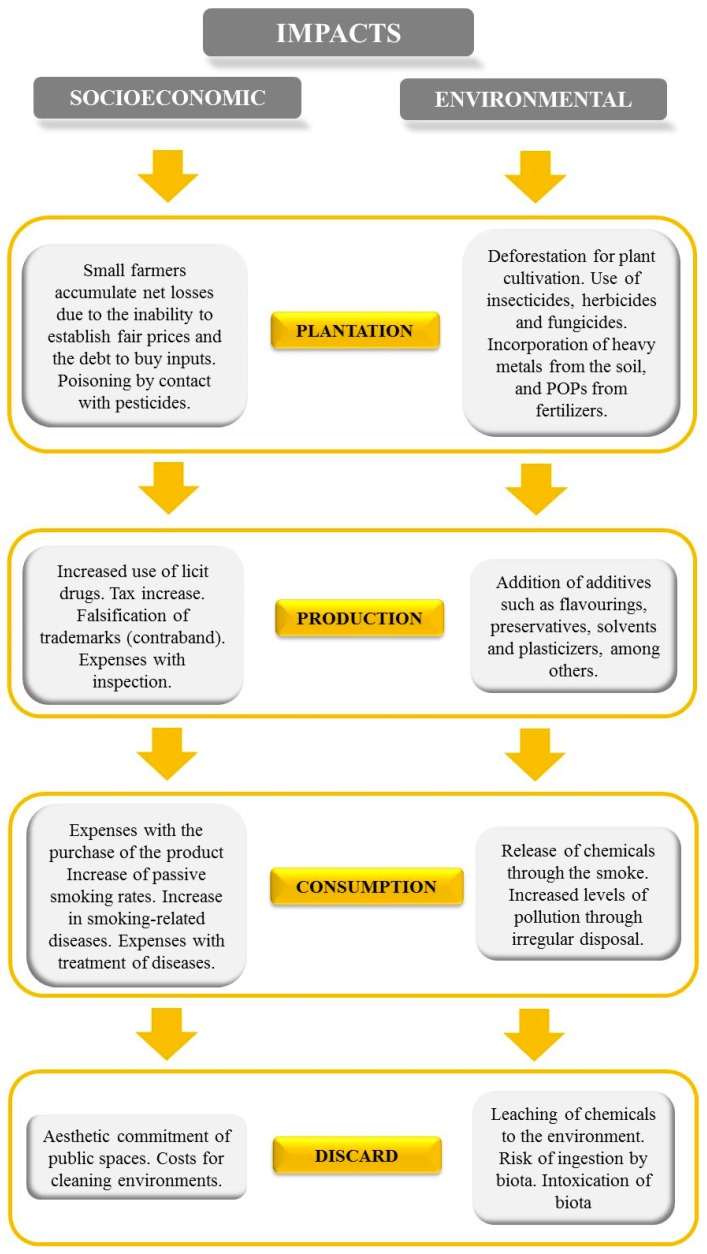
Socioeconomic and environmental impacts generated during the different phases of tobacco plantation, production, consumption (smoking) and the discarding of its wastes.

The effects of cigarette smoking on active and passive smokers represent a serious public health issue, widely assessed and discussed worldwide. However, smoking is also related to a number of situations that generate other serious consequences. The production and consumption of cigarettes and the irregular disposal of cigarette butts (filter after smoking, with or without burned tobacco remnants) involve a series of large and relevant impacts, thereby affecting various socioeconomic and environmental aspects (Figure 1).

The integrated system of tobacco production in cigarette industries is a trap imposed on family farmers [19]. The supposed sales certainty and support offered by tobacco companies are nothing but an illusion. The industries (most of them multinational companies) involved make profits, and family farmers only have losses, either because the minimum price agreed in advance penalizes the producers, or because an increase in production also leads to an increase in the losses of tobacco growers.

Tobacco cultivation and curing are directly responsible for deforestation, given that forests are eliminated and replaced with tobacco plantations, and the wood is burned to cure the leaves of the plants [20]. It is estimated that 11.4 million metric tons of wood are annually required for curing tobacco. After production, more wood is used to produce the packaging material used in the final product [21].

The deforestation required for the cultivation of the plants that produce tobacco brings a series of associated impacts; however, the cultivation phase is even more critical. The intensive use of pesticides contributes to pollution and degradation of water and soil [22]. According to Slaughter et al. [3], the United States Environmental Protection Agency determines which pesticides can be used in tobacco crops, as well as how they should be used; however, it does not regulate pesticide residues in tobacco. The United States Department of Agriculture has found that these wastes exceed the current residue limits considered safe for human and environmental health.

The working conditions of tobacco growers expose them to occupational hazards. The health of these farmers is systematically damaged in several ways, i.e., by the use of agrochemicals, by direct contact with the moist plant that releases nicotine (absorbed by the epidermis), and by the smell of the leaves during drying in the greenhouses [23,24,25]. The contact with green tobacco leaves is responsible for a type of poisoning known as “green tobacco sickness”. This disease is caused by the dermal absorption of nicotine and produces a number of symptoms, such as dizziness, trembling, weakness, and nausea [26]. The level of nicotine in the blood of those who work in tobacco plantations is about the same or even many times higher than that found in smokers [19]. 

Regarding agrochemicals, the products used are of high toxicity, such as organophosphates, carbamates, and pyrethroids. Organophosphate and carbamates insecticides are powerful inhibitors of enzymes that are essential for the proper functioning of the nervous system. These substances may be absorbed into the body through contact with skin, swallowing, or inhaling. They influence the central nervous system, blood, and other organs. The pesticides composed of pyrethroids are absorbed through the digestive tract and respiratory and cutaneous routes. They cause mucosal irritation and bronchial asthma [27]. The situation is aggravated by the lack or inadequate use of personal protective equipment, lack of information about how to handle pesticides, and improper disposal of packaging [23,24]. In addition to directly affecting the farmers, pesticides affect the soil and the water, extending the effects beyond human health.

In addition to the direct issue related to health problems affecting both farmers and smokers, there is also a relevant issue involving natural environments, which are strongly impacted by the presence of remnants of smoked cigarettes, composed of filters with or without tobacco residues, which are improperly discarded. Smokers often discard cigarette butts in public places improperly [14,28,29,30,31]. The prevalence of this habit is associated with several factors, such as poor law enforcement regarding littering, in general; absence of adequate penalties; and poor advertisement of the environmental problems caused by tobacco products, despite the focus of public campaigns on health issues for both smokers and second-hand smokers. A serious lack of vision of the entire tobacco use time line, its products and their resulting wastes makes anti-tobacco actions and pollution control difficult [7].

It is known that, in more developed urban centers, especially in areas with a high concentration of users, there are usually a large number of cigarette butts discarded by smokers in public spaces, such as roads, commercial areas, and bars. Probably, this fact is due to the greater concentration of points of sale of the product in these places [1,32,33,34]. This scenario has been found in central areas of Mar del Plata (main tourist city of the Argentine coast) by studies whose main goal had been to determine whether the level of use of certain areas had an influence on the production of waste. One of those studies compared four areas, of which three were intensively used. The authors found a total of 20,336 items, of which 33% were cigarette butts. The total amount of cigarette butts found in the three areas of leisure and intensive commercial use was 5897, in contrast to the total of 820 cigarette butts found in the less crowded area [32]. Another study compared waste production in three different locations, two areas with intense nightlife (commercial center, restaurants, pubs, etc.) and another more isolated residential area with practically no nightlife. A total of 13,503 items were found, 42.8% of which were cigarette butts (5575 items in areas with intensive nightlife, and only 201 in the residential area) [33]. According to the authors, part of the waste observed in the study might have been one of the sources of waste found on beaches along the coastline of the city.

In addition to the urban centers of large cities, cigarette butts are also found in other places. For example, large amounts of these waste items can be easily found in the sand of countless beaches around the world [35,36,37,38,39]. Clean-up activities performed on beaches around the world, coordinated by Ocean Conservancy (a United States-based conservation organization) (Ocean Conservancy: International Coastal Cleanup) and conducted by volunteers, confirm the magnitude of the pollution caused by cigarette butts in these environments. In the 2016, 2017 and 2018 reports, for example, this type of waste occupied the first position in the ranking of the number of waste items collected, with amounts greater than 1,800,000 items annually found. Probably, waste materials improperly discarded in cities are carried by wind and/or water runoff in the drainage system, reaching coastal areas [40,41]; or these materials are deliberately discarded on the beaches by users of these environments. Cigarette butts are small and light (sometimes sand-colored). Therefore, these items remain in the sand, regardless of cleaning efforts, possibly being buried over time.

In the environment, cigarette butts have become the source of a wide variety of toxic wastes. Some of these wastes already naturally occur in the environment at low concentrations (e.g., arsenic, heavy metals, nicotine) and others are synthetic, such polycyclic aromatic hydrocarbons. Both types may be transferred through solution after leaching into the soil [3,4,42,43]. When these compounds are in the environment, they contaminate the soils, and can be carried by rainfall to aquatic environments, where they can be detected [1,13,43,44,45,46,47]. There are some reports about the effects of these compounds on aquatic biota, mainly concentrated in invertebrates and few fish species [3,43,44,48,49,50,51]. But even then, the risks posed by cigarette butts to the aquatic biota remain underestimated because both their qualitative and quantitative aspects are poorly known [7].

In addition to being a vector for the transport and input of toxicants into aquatic habitats, cigarette butts also pose a physical risk to the aquatic biota. Tobacco-derived environmental contamination is usually more valued when affecting fauna. For example, Macedo et al. [52], reported the presence of cigarette butts in the digestive tract of two species of sea turtles (*Chelonia mydas* e *Eretmochelys imbricate*) collected along the northeast coast of Brazil.

Unfortunately, there are only limited scientific studies that report the negative interactions between tobacco smoking wastes and plants. Since cigarette butts are typically soil contaminants, the leaching of toxic substances makes them available to the soil and its communities. In this context, the transfer of nicotine from discarded cigarette butts into other plants cannot be neglected [53,54]. For instance, the “horizontal transfer of natural products” and its relevance for contaminations of plant-derived commodities have to be considered [55,56].

Other residues used in the packaging of cigarettes—such as paper, ink, cellophane, aluminum, and glue—also reach the environments in the form of irregular waste. They can cause other damages in addition to those related to the effects of the filters on the environments and biota [57].

The World Health Organization (WHO) Framework Convention on Tobacco Control [58] recommends the Parties to observe guidelines from its experts working groups that coalesce knowledge based on sound scientific literature in order to abate and improve all issues generated by tobacco smoking. For instance, Articles 17 and 18 of its main most recent directives suggest that sellers should be offered technically and financially viable alternatives to deal with their wastes; thus extending the responsibility for tobacco products and waste control to the environment as much as to people [7].

Throughout the world, numerous initiatives have been established to reduce the presence of cigarette butts in environments. The most frequent measures used have been the ban on smoking in some public places [59,60,61,62,63], the distribution of mini ashtrays for individual use [64], and cleaning actions with the participation of volunteers [65]. 

Some initiatives have involved recycling of cigarette butts [66] to prepare different paper sheets, using cellulose acetate without previous treatment and cigarette butts cooked in an alkaline medium. The resulting product could be interesting to the recycled paper industry either in a pure form or mixed with ordinary paper pulp in different amounts, depending on the requirements of the final product. In addition, some commercial initiatives also exist that try to use cigarette butts. Brazil (Bituca Verde, or Green Butts and Renova Ambiental, or Renew Environment), France (GreenMinded, works with a French processing plant, MéGO) and the United States (Terracycle, a New Jersey-based firm) all have examples of entrepreneurship with cigarette butts recycling. However, all of these actions are palliative and do not address the source of the problem.

Cigarette butts are a disputable waste for recycling. It is considered a hazardous waste, and from some points of view “unrecyclable”. All possible steps in its recycling can be per se a new socio-environmental threat. To be recycled, they need to be collected from special containers or from the ground, exposing workers. Its pre-treatment will, necessarily involve transportation, stocking, energy and chemicals consumption. The resulting cellulose that is the target product will be contaminated, as well as any effluent or waste from the process, demanding for difficult and possibly expensive disposal. In this way, cigarette butts are the waste of a hopeless non-circular process.

## 3. Conclusions

The problems generated by smoking, both from the socioeconomic and the environmental points of view, have become an important and emerging issue, demanding joint efforts and actions focused especially on the reduction of environmental impacts. These aspects have been still little assessed.

Effective mitigation of problems requires four main lines of action. The first would be the control and supervision of the tobacco production process, especially during planting and handling of the leaves, in order to minimize the contamination of nearby water resources, and protect the health of the farmers by demanding the use of personal protective equipment. The second would be the unrestricted ban on smoking in all public spaces, including beaches, imposing fines in cases of noncompliance with the legislation. This action could be an effective measure for reducing the risk posed to passive smokers and the production of waste, thus minimizing impacts on the biota. The third action would be the promotion of educational campaigns not only addressing the risks for smokers (active and passive) and the environment, especially the aquatic environments and their biota, but also warning about the economic costs incurred with cleaning the environments and treating smoking-related diseases. Finally, it would be necessary to increase taxes on cigarette sales, in order to discourage consumption and combat smuggling.
